# Biodiversity and Constrained Information Dynamics in Ecosystems: A Framework for Living Systems

**DOI:** 10.3390/e25121624

**Published:** 2023-12-05

**Authors:** Kazufumi Hosoda, Shigeto Seno, Rikuto Kamiura, Naomi Murakami, Michio Kondoh

**Affiliations:** 1 RIKEN Center for Biosystems Dynamics Research, 6-2-3 Furuedai, Suita, Osaka 565-0874, Japan; riku.kimura@riken.jp (R.K.); nmurakami703@gmail.com (N.M.); 2 Center for Information and Neural Networks (CiNet), National Institute of Information and Communications Technology (NICT), Osaka 565-0871, Japan; 3 Institute for Transdisciplinary Graduate Degree Programs, Osaka University, 1-5 Yamadaoka, Suita, Osaka 565-0871, Japan; 4 Life and Medical Sciences Area, Health Sciences Discipline, Kobe University, Tomogaoka 7-10-2, Suma-ku, Kobe, Hyogo 654-0142, Japan; 5 Graduate School of Information Science and Technology, Osaka University, 1-5 Yamadaoka, Suita, Osaka 565-0871, Japan; senoo@ist.osaka-u.ac.jp; 6 Graduate School of Life Sciences, Tohoku University, 6-3 Aoba, Aramaki, Aoba-ku, Sendai 980-8578, Japan; michio.kondo.b8@tohoku.ac.jp

**Keywords:** synthetic ecosystem, experimental ecosystem, microcosm, information, entropy, diversity, adaptability, adaptation, evolution, homeorhesis, homeostasis, constraints

## Abstract

The increase in ecosystem biodiversity can be perceived as one of the universal processes converting energy into information across a wide range of living systems. This study delves into the dynamics of living systems, highlighting the distinction between ex post adaptation, typically associated with natural selection, and its proactive counterpart, ex ante adaptability. Through coalescence experiments using synthetic ecosystems, we (i) quantified ecosystem stability, (ii) identified correlations between some biodiversity indexes and the stability, (iii) proposed a mechanism for increasing biodiversity through moderate inter-ecosystem interactions, and (iv) inferred that the information carrier of ecosystems is species composition, or merged genomic information. Additionally, it was suggested that (v) changes in ecosystems are constrained to a low-dimensional state space, with three distinct alteration trajectories—fluctuations, rapid environmental responses, and long-term changes—converging into this state space in common. These findings suggest that daily fluctuations may predict broader ecosystem changes. Our experimental insights, coupled with an exploration of living systems’ information dynamics from an ecosystem perspective, enhance our predictive capabilities for natural ecosystem behavior, providing a universal framework for understanding a broad spectrum of living systems.

## 1. Introduction

Living systems can be perceived as systems that convert energy into information. The increase in the biodiversity in ecosystems is seen as one of the conversion processes. Therefore, understanding ecosystem dynamics from the perspective of information, the focus of this special topic is important for both preventing ecological crises and grasping the fundamental nature of living systems. While it is argued that organisms increase their systemic information through “adaptation by natural selection,” ecosystems lacking overt natural selection mechanisms require a distinct framework to understand phenomena that appear to enhance their information. “Adaptability” is posited as a concerted counterpart to natural selection, embodying the essence of information processing in living systems [1]. From the viewpoint of ecosystem framework, modeling living systems has propelled our comprehension of how they augment their information by increasing the entropy of the universe [1,2]. In systems ecology, numerous measurable macroscopic parameters encompassing information have been introduced [3]. Theoretical ecology has identified pivotal challenges [4] and offered various mechanisms addressing them in the context of adaptation [5,6]. Despite these advances, a comprehensive quantitative understanding of the dynamics of ecosystems and broader living systems remains elusive.

The interdisciplinary nature of this field presents challenges in fostering idea exchange among researchers, potentially impeding progress [7]. A contributing factor may be the need for explanations that are more extensive than those typically found in ordinary papers. This study, which centers on the changes in ecosystems, approaches a wide array of living systems from the perspective of an ecosystem framework. Aligned with the goals of this special issue—to encourage interdisciplinary dialogue—this study offers both a thorough and accessible introduction as well as preliminary experimental findings for greater unseen ideas. The extended introduction is designed to elucidate the relevance of the experiments conducted in this study, demonstrating their significance not only for understanding ecosystems but also for providing insights into a broad spectrum of living systems.

### 1.1. Ecosystem Framework and Macroscopic Parameters

Grasping the overall changes in ecosystems through the lens of macroscopic parameters, such as entropy and information, is beneficial for comprehensive understanding. If the alterations within various ecosystems can be encapsulated by a limited set of macroscopic parameters, it not only facilitates predictive modeling but also indicates the presence of robust constraints, effectively reducing the substantive dimensionality of the systems.

While this approach diverges from the conventional definition of “ecosystem,” expanding the concept of ecosystems to encompass lifeless environments allows for a seamless integration from molecular to ecosystems. Often, even in typical ecosystems, boundaries are indistinct and defined abiotically. To circumvent confusion with ecosystems, we introduce the term “panecosystems” to describe systems that include ecosystems but also extend to contexts devoid of living entities, thereby enabling analysis through an ecosystem framework. To fully comprehend the significance of ecosystem stability and its underlying mechanisms, it is imperative to consider the entropy or information within these panecosystems.

**Figure 1:**
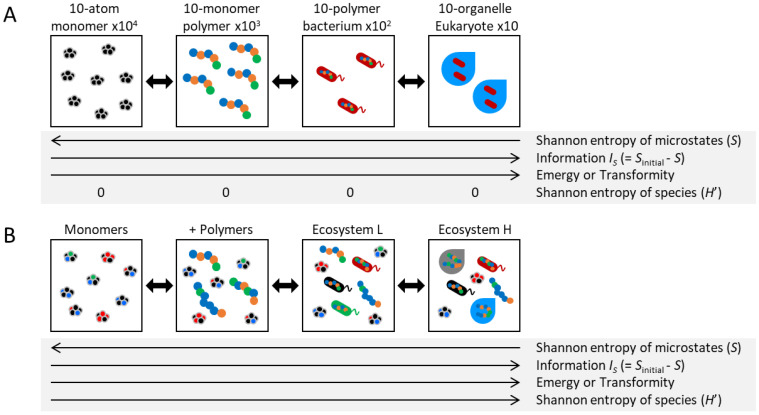
Ecosystem framework for a wide range of living systems. (**A**) Four panecosystems with an emphasis on the spatial arrangement of an equivalent number of identical atoms in a simplified scenario that excludes other variables, such as chemical energy. (**B**) Panecosystems that are marginally more realistic than those in A incorporate a diversity of components. The three types of macroscopic parameters associated with information—*I_S_*, Emergy, and *H′*—although distinct in definition, would roughly exhibit higher values in the systems depicted on the right side. Refer to the main text for detailed explanations.

Let us consider, as a hypothetical exercise far removed from practical reality, the four panecosystems depicted in Figure 1A. These are represented by focusing solely on the spatial distribution of an equal total number of identical atoms in a scenario where other factors, such as chemical energy, are disregarded. In the monomer system (at the leftmost end of Figure 1A), each monomer possesses a degree of freedom in its state (position, velocity, etc.). This macrostate encompasses numerous microstates. Statistical entropy (*S_B_*) has been defined as a quantifier of the number of possible microstates (*W*) expressed as $S_{B}=k_{B}\;lnW$, where *k_B_* is the Boltzmann constant. Note that understanding formulas is not essential to grasp the concepts presented in this study. In the polymer system (positioned to the right of the monomer system in Figure 1A), defining the state of one monomer inherently limits the potential states of the remaining nine monomers within a certain proximity. As a result, the polymer system demonstrates greater order and constraints relative to the monomer system, manifesting in a reduced number of possible microstates or a lower statistical entropy. A similar trend of diminishing statistical entropy can be observed when moving rightward in Figure 1A.

This entropy, *S_B_*, is commonly understood to be analogous to the equilibrium case of Shannon entropy (or expected information) for microstates *x*, expressed as $S_{H}=-\sum_{x}p\left(x\right)lnp(x)$, where *p*(*x*) is the probability of state *x*. This relationship can also be extended to non-equilibrium states [8,9,10,11]. Entropy is recognized not only as an indicator of the direction in which a state will evolve but also as a fundamental link to information, energy, and work. In this study, we do not dwell on the distinctions but rather adhere to the prevailing conventions of information thermodynamics. We employ *S_H_*, which is applicable to non-equilibrium states, as a surrogate for entropy (*S*) in the broader contexts of thermodynamics and statistical mechanics.

The amount of system information could be simply defined as the decrease in the number of possible microstates, i.e., the difference in statistical entropy [1]: *I_S_* = *S*_initial_ − *S*. This metric interprets the extent to which states are constrained by order. In this context, as the statistical entropy decreases as one proceeds rightward in Figure 1A, the information correspondingly increases. This definition of information is simple, intuitive, and theoretically convenient as it directly relates *S*, despite the unclear initial state *S*_initial_. Therefore, in this study, “information” means *I_S_* unless otherwise specified. However, their practical application to complex systems presents significant challenges, and actual measurement is fraught with difficulties [1]. Furthermore, note that this definition is sometimes not appropriate when considering information about living systems. For example, if all internal components in the system disappear, *S* will be zero, and *I_S_* will be maximum. Therefore, more suitable definitions of information exist, contingent upon the specific situation. For instance, the relative entropy between the current state and the steady state is thought to exemplify the concept that ecological communities acquire information from the environment as they near equilibrium [12]. It has also been demonstrated that relative entropy can depict state stability in models of the adaptive dynamics of ecological communities [13].

In the field of systems ecology, information is addressed as the “components and connections of system organization” [3]. This definition intuitively aligns with the aforementioned concept of *I_S_*. Although translating this definition into mathematical terms poses a challenge, systems ecology proposes some measurable indexes that include the concept of information, such as “E**m**ergy” [3,14,15]. This term represents the summation of energy required to generate a system and has been utilized as an indicator of ecosystem sustainability. Intuitively, incinerating 50 kg of humans or bacteria may yield a similar amount of energy. However, the creation of a 50 kg human would consume more energy. This discrepancy suggests the involvement of information, positioning Emergy as a substantive macroscopic parameter encapsulating information. Moreover, the concept of “Transformity” is defined as Emergy divided by available energy, potentially drawing it closer to the concept of information.

Distinct from the information *I_S_*, the Shannon–Wiener index, frequently employed as a diversity index within ecosystems [16], denotes the Shannon entropy of species [17] as $H^{\prime }=-\sum_{i}p_{i}lnp_{i}$, where *p_i_* is the proportion of individuals belonging to the *i*-th species. This index is zero for all panecosystems in Figure 1A involving only a single species or type. It is important to note that while various terms with “information”, “entropy”, and “diversity” are prevalent, there are both nuanced similarities and even conceptual inversions between them.

However, adopting a slightly more realistic perspective with diversity, as depicted in Figure 1B, the diversity index *H′* increases as one moves to the right, aligning with the direction of *I_S_*. This co-direction suggests that the constraints by the “realistic perspective” of the living system somehow connect *H′* and *I_S_*. In other words, understanding these relationships will directly lead to an understanding of living systems. Though diversity takes various forms, it tends to align with the direction of *I_S_* in reality. Diversity’s relationship with thermodynamic indicators like *I_S_* or Emergy is typically more tenuous, yet it is often more convenient and allows for more straightforward measurement. No single form of diversity would be inherently superior, and even combinations of various diversity indexes lead to a dramatic reduction in system dimensions.

Ecological studies have characterized ecosystems using various diversity indices [18,19,20]. For instance, Species Richness, which simply counts the number of different species in an ecosystem, is the most intuitive and commonly used measure of biodiversity. However, this indicator does not account for species abundance, rendering it a limited expression of biodiversity and a challenging metric to estimate accurately from natural observations. Consider an ecosystem with three species, each comprising 1000 individuals, totaling 3000. Contrastingly, another ecosystem might have 2997 individuals of one species and one individual from each of three species, totaling 3000 individuals across four species. While the latter demonstrates greater Species Richness, the presence of a single individual species may have minimal impact on the ecosystem’s characteristics. Moreover, observing a solitary individual is highly probabilistic, and Species Richness is greatly influenced by the scope of observation. The above-mentioned *H′* index is a diversity measure that incorporates the likelihood of encountering individuals based on probability theory [18]. The Hill number is a more generalized indicator that enables multi-faceted evaluation, used in the Results and Discussion section below. Utilizing profiles with multiple indicators is considered preferable to selecting a single or limited measure [20]. It is also crucial to consider not only species differences but their phylogenetic disparities and functional diversity [19]. Functional diversity, in particular, is key to comprehending ecosystem processes and their responses to environmental stresses and disturbances, marking a rapidly evolving research area. As stated, there are various diversity indicators, each with its merits and limitations, and the field continues to grow with new insights and measures. These can be regarded as realistic macroscopic parameters of ecosystems.

Those macroscopic parameters, such as *S* and *I_S_*, Emergy, Transformity, and various diversity indexes, are applicable beyond ecosystems. Applying them to a wide range of living systems as panecosystems seamlessly will highlight the characteristics of each system and provide an integrated understanding of living systems.

### 1.2. Information Dynamics in Living Systems: Macroscopic and Microscopic Perspectives

How do living systems accumulate information? As depicted in Figure 1B, according to the Second Law of Thermodynamics, in an isolated system—one devoid of external material or energy inputs—the entropy *S* would increase (signifying a movement to the left in the figure), indicating a decrease in information, and ultimately reaching a state of equilibrium. However, when considering living systems, it is important to note that even Earth is not an isolated system but rather a closed system. In the context of these living systems, the universe represents the sole example of an isolated system. Thus, living systems, being subject to external energy inputs, have the capacity to increase information. This process does not contradict the Second Law of Thermodynamics as long as the increase in information within living systems is offset by an overall increase in entropy within the universe.

Nonetheless, it is not a given that energy input always increases information. For instance, simply raising or lowering the system’s temperature or altering its volume by expansion or contraction would not typically result in a continuous growth of information. The continuous information growth necessitates “agents” capable of information processing, akin to Maxwell’s demon [21] that can manipulate internal components. In ecosystems, the organisms residing internally serve as agents capable of processing information. This ability is not limited to higher organisms, such as humans; even bacteria possess systems enabling them to respond optimally to their environment [22].

In the case of organisms with explicit self-replication capabilities, it can be posited that those with higher informational content may have enhanced survival prospects through mutation and natural selection. However, this does not necessarily imply an average increase in the system’s informational content; rather, it may lead to a decrease in information among systems that are not selected. Moreover, it is also difficult to grasp the reproduction or disappearance of the system itself in a reversible manner.

By considering systems at the unit level of ecosystems like panecosytems and addressing processes like polymer synthesis, cellular replication, and their respective reverse reactions through an information thermodynamics analysis, conditions for self-replication have been mathematically formulated from a statistical entropy standpoint [23]. This framework suggests that if the system changes in a way that increases the entropy of the universe more effectively, then the energy input to the system will result in a generation or enhancement of the system information. It can be used to explain how pre-living molecular systems gained the function of self-replication or how the Earth has given rise to a variety of species. In other words, considering the time scale of billions of years or infinity, it might be possible to think that it is no coincidence that Figure 1B moves to the right. However, this framework has not yet provided dynamics in a specific timescale and upper limits, such as the maximum amount of information, or eternal stability, such as immortality.

Regardless of whether it is a simple chemical reaction system or a complex living system, the behavior of the equilibrium state can be described using free energy, incorporating both energy and statistical entropy. However, in a non-equilibrium state, various dynamics can occur in high-dimensional complex systems, making them extremely difficult to understand and predict. Moreover, the emergence of sequence information in polymers such as DNA and proteins further complicates the analysis.

In order to understand the characteristics of information carriers or other specific hardware, the interaction mechanism of elements within a system, and the corresponding dynamics on a specific time scale, it is necessary to consider not only the macroscopic perspective but also the mechanism of microscopic dynamics.

As for information carriers, speaking broadly, the simple answer for organisms would be genomic DNA, although it is known that genomic DNA alone is insufficient to represent the heritable information [24,25,26]. However, for more general living systems, it is useful to consider the process by which information carriers are born in dynamics. Theoretical research has revealed that competition in two hierarchical layers, intracellular and intercellular, make two originally symmetrical elements asymmetrical into “information carriers,” which is not directly functional and become an information source for the functional units, and “functional units,” which is directly functional and does not become information source [27]. This research shows that the characteristics of each element become differentiated regardless of their original characteristics. Thus, the framework can be applied not only to the differentiation of DNA and proteins, i.e., the origin of the central dogma but also to cell differentiation or the division of roles in social animals. Extended to an extreme, it may be possible to consider that two system parameters acquire characteristics appropriate for the roles of information carriers, e.g., robust and not directly functional, and functional units, e.g., flexible and directly functional, regardless of their original characteristics. This assumption may provide clues to elucidate the information carrier of ecosystems in experiments in this study.

The fidelity of replication of sequence information in organisms has been extensively studied in the context of the “error catastrophe” concept, wherein excessive copying errors can lead to inviability [28]. While this concept primarily addresses a high level of accuracy in genetic self-replication, it might be valuable when considering other systems, such as ecosystems.

The perspective of the dynamics of sequence information also has features in common with other systems, such as ecosystems. Theoretically, the synthesis and destruction process of polymer sequence information has been analyzed from the perspective of the hardness of the processes [29], which would be similar to the concept of Emergy in the ecosystem mentioned above. Also, it is known that slow kinetic synthesis produces complex polymer sequences [30], which would be related to the trends of ecosystems in that developed ecosystems are slow [31] and that mutualism and diversity are enhanced in slower environments [32]. From this microscopic dynamic perspective, there arises a potential for macroscopic parameters that can describe the stability of conditions and the direction of change from molecules to ecosystems.

### 1.3. Diversity and Information Dynamics in Ecosystems: Necessity of Adaptability

From the information thermodynamics perspective above, it may be natural that ecosystems with higher diversity are more stable at Earth-level sizes and very long timescales. However, understanding the human-level time scale of each ecosystem would require more specific mechanisms. Note that the consideration below ignores details and roughly assumes that more complex or diverse systems have a larger amount of information.

The relationship between ecosystem stability and diversity is a paramount topic in ecology. Ecosystems are posited to develop towards a stable state and accrue information following significant disturbances [31]. Empirical observations have led to the hypothesis that complex ecosystems tend to be more stable [33]. Conversely, mathematical models indicate that as the number of species increases in simple random networks, the stability correspondingly diminishes [4]. This principle holds across networks of various structures, not limited to random configurations. However, network models that include adaptation [5] or network assembly models by adding new species, i.e., not organism-level adaptation but ecosystem-level adaptation [6], can exhibit enhanced stability with growing diversity. These insights suggest that “adaptation” is a key when considering ecosystem diversity and information dynamics.

It is necessary to consider this “adaptation” in ecosystems more deeply. First, in the genetic adaptation of natural selection, organisms are systems that are selected. The system information does not consistently increase, and many systems disappear. However, if we consider the population as a panecosystem, it is possible for the system to consistently increase information. In other words, as living organisms act as information-processing agents through natural selection, even if many individual organisms disappear, the population can consistently increase in information. However, this is true within one population and does not necessarily increase the information of ecosystems with diverse populations.

Natural selection alone is not sufficient to explain the increase in ecosystem information. Ecosystems, unlike organisms, do not have clear boundaries or solid information carriers and, therefore, are not subject to sophisticated selection. In the ecosystem itself, it is impossible to randomly make various copies and end up with the best ones remaining. Note that in the framework of natural selection, adaptation and fitness are determined post hoc. While fitness is often predefined for each organism in theoretical studies, it actually varies depending on the environment or situation. Therefore, fitness cannot be definitively established before the specific environment or situation is encountered. Similarly, adaptation is fundamentally the result of these environmental interactions. Thus, what is required for the ecosystem per se is not only a serendipitous adaptation by processing results but also a successful adjustment that preserves and increases information even in the face of all unexpected disturbances, i.e., “adaptability.” In this study, we adopt the definition of adaptability in the previous study [1] as “the ability of a system to cope with unexpected disturbances in the environment.”

Adaptability can be thought of as the ability of a system to use energy to retain or increase information. The concept of the distinction between adaptation and adaptability does not imply that organisms only adapt due to natural selection, but organisms also have adaptability [1]. Natural selection was not considered the only mechanism for species modification even when it was proposed [34]. The adaptability of organisms encompasses adaptive phenotypic plasticity in response to unforeseen situations that is indeed observed in microbial experiments [35,36] and is considered to be an efficient exploratory dynamical process inherent even in cells and organisms [36]. Nevertheless, the mechanism that achieves adaptability and information increase in ecosystems remains unclear.

### 1.4. Mechanism for Information Increase and Identifying Information Carriers in Ecosystems

**Figure 2:**
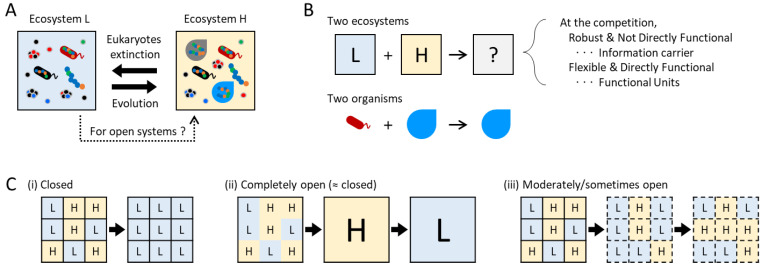
Mechanism for information increase and identifying information carriers in ecosystems. (**A**) The relationship between ecosystems L and H is depicted in Figure 1B. (**B**) Competition between two systems by coalescence is an extreme case of interaction. Upper and lower show the cases of ecosystems and organisms, respectively. The strategies for inferring information carriers and functional units are summarized on the right. (**C**) One simple mechanism by which ecosystems sustain or increase their information. (**i**) “Closed” refers to scenarios where there is no interaction between different ecosystems. (**ii**) “Completely open” describes situations in which boundaries between different ecosystems disappear. (**iii**) “Moderately/sometimes open” denotes cases where some level of interaction occurs between different ecosystems, though they are not entirely mixed, and boundaries between them still exist. Alternatively, this could imply that while in most instances there is no interaction between different ecosystems, occasionally they become completely intermixed. Explanation is provided in the text.

When considering a mechanism for information increase in ecosystems, other than natural selection, it would be easiest to first consider some kind of “competition between ecosystems” as an information selection. Figure 2A shows the relationship between ecosystems L and H, depicted in Figure 1B. If these ecosystems were a closed system, a shift to the left could occur rapidly by species extinction, while a shift to the right would necessitate a long time for evolution. If the spatiotemporal scale is limited, unlike Earth, and evolution is negligible, ecosystem L would be more stable, and information would decrease. However, because ecosystems are open systems, the reintroduction of extinct species from external sources is feasible, making a shift to the right possible. This leads to important discussions about the competition between which information will remain when two ecosystems interact.

Consider competition between two ecosystems when the ecosystem is open. At the extreme, this is a question of what kind of information will result when the two are mixed (Figure 2B). Although complete coalescence is unrealistic in natural ecosystems, similar phenomena are likely commonplace at the boundaries between distinct ecosystems. This competition by coalescence is not like natural selection as a competition within a species population. Rather, in an organism-level analogy, it would be like competition between species, i.e., what happens when two organisms exist in the same place. In a predator–prey relationship, information from the prey, primarily utilized to sustain the predator’s information, would diminish. In the case of symbiosis, both information would remain, resulting in merged information.

Next, consider the competition between ecosystems H and L in a meta-ecosystem consisting of multiple ecosystems H and L (Figure 2C). Let us assume that ecosystem H is stronger in competition than ecosystem L and that when those interact, ecosystem L becomes H. If every ecosystem is completely closed and there is no interaction, all ecosystems H become L, as discussed above (Figure 2C(i)). Conversely, if every ecosystem is completely open, i.e., the meta-ecosystem becomes a single merged ecosystem, the merged ecosystem once becomes ecosystem H, but it eventually becomes ecosystem L, assuming the scale difference between the meta-ecosystem and each ecosystem is negligible (Figure 2C(ii)). Only if the ecosystem is moderately open can information on ecosystem H be preserved (Figure 2C(iii)). In natural ecosystems, for instance, this moderately open scenario might correspond to a meta-ecosystem separated by rivers that occasionally intermix due to relatively rare events such as typhoons. Alternatively, simply, it might be that the rate of transition between ecosystems is low, but this was not experimentally confirmed in this study. In this way, ecosystem information can be preserved through ecosystem competition at moderate openness.

It is possible to infer the information carrier and functional unit of ecosystems if it is indeed possible to maintain or increase information through such competition between ecosystems. As discussed above, let us assume that the two system parameters differentiate into those that are robust and not directly functional and those that are flexible and directly functional, as the information carrier and functional unit, respectively, regardless of their original characteristics. Then, simply examining what parameter is more robust or flexible during the competitions may provide clues to identify the two roles, even without knowing their properties.

This method of identifying information carriers is not surprising when considering a typical example. One of the most notable examples of adaptability in living systems is the brain, which utilizes energy for information processing. Let us consider two parameters in the brain: synaptic weight and neural activity. When mathematically modeled as artificial neural networks, these are often expressed as *w* and *x* vectors, respectively, and function as *w∙x* [37]. Thus, the characteristics of those two parameters are often symmetrical. However, there is a clear difference between the two parameters in both the brain and artificial neural networks: synaptic weight and neural activity are more robust and flexible as information carriers and functional units, respectively. In artificial neural network models, synaptic weights are developed gradually through a learning process. Often, these weights remain unchanged when the model is utilized for inference. Therefore, synaptic weight can be regarded as an information carrier that defines the model’s characteristics. In contrast, neural activity is a variable that changes at each instance of inference. It is a functional parameter that varies in response to input and generates output. Consequently, in artificial neural networks, synaptic weight and neural activity can be interpreted as information carriers and functional units, respectively. While it cannot be claimed that these are identical to the brain, artificial neural networks were initially devised as brain models and are believed to replicate similar characteristics.

Additionally, there are some similarities between ecosystems and the brain, as both are living systems that use energy to process information without natural selection [38]. A typical example of the brain’s adaptability to respond to unexpected situations is inspiration with the Eureka effect, which is an ability to come up with the correct answer to unlearned tasks without any learning by taking a relatively long time to think [39,40]. Also, the brain can learn a new concept from one example of Hebbian learning, a simple process of strengthening the synaptic connections that are used [41]. Conversely, it is theoretically shown that ecosystems can have adaptability akin to Hebbian learning in neural networks [42]. This notion of adaptability as an inherent characteristic of living systems also aligns with the “free energy principle,” which assumes that brains and organisms inherently predict their state, ensuring their survival by minimizing the “prediction error” using sensory feedback from the environment [43]. This convergence suggests that both organisms and ecosystems are inherently grounded in principles of adaptability, ensuring resilience in the face of uncertain disturbances.

### 1.5. Freedom and Constraints, Homeostasis and Homeorhesis

Adaptability, which is an essential ability of living systems, is thought to require a balance between freedom and constraints. It would be reasonable for information-processing systems to have strong orders and keep large amounts of information, that is, constraints of the system states in the context of statistical entropy. On the other hand, responding to unexpected disturbances requires not only constraints but also freedom. Thus, a balance between the opposing facets of freedom and constraint is considered to be a fundamental demand on living systems [1,44].

The property that expresses the balance in an easy-to-understand manner is “homeorhesis” [45]. Homeorhesis is a property of a dynamical system that maintains a particular trajectory despite perturbations from the environment. This is similar to homeostasis, which describes the property of maintaining a stable state [46], but it is different in that a system with homeorhesis is constantly changing. It should be noted that living systems exhibit characteristics that can be identified as both homeostasis and homeorhesis, and it is not feasible to distinctly differentiate between these two in actual phenomena. These terms merely serve as useful concepts for emphasizing a particular characteristic of a dynamic system.

**Figure 3:**
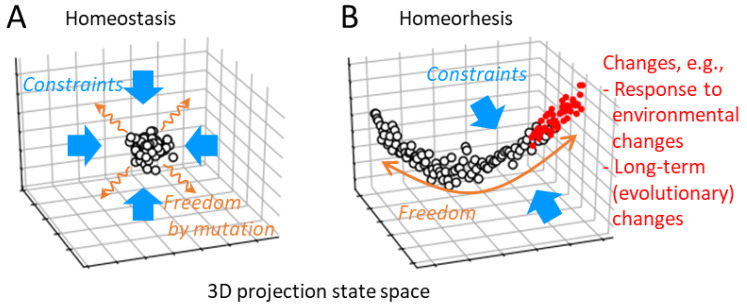
Freedom and constraints, homeostasis and homeorhesis, in dynamics of living systems. The state of a high-dimensional living system is depicted in 3D projection state space. Open circles indicate variation in the state space due to fluctuation. (**A**) Adaptation through natural selection. (**B**) Homeorhesis and Dominant Mode Hypothesis. Red closed circles indicate the changes, such as response to environmental changes or long-term changes.

To discuss the necessity of homeorhesis, let us consider, conversely, that adaptation through natural selection is possible even without homeorhesis. In adaptation through natural selection, freedom and constraint can be provided to the organisms independently (Figure 3A). First, organisms need a stable phenotype that corresponds to a static information carrier for being selected by the environment. This is homeostasis, which is a property of system dynamics as constraints. On the one hand, freedom in variation is required for the adaptation, which is provided as random mutations, independent of the properties of the dynamics. Therefore, as a property of dynamics, as long as there is homeostasis to maintain a stable state, adaptation through natural selection is possible.

On the other hand, in the case of an ecosystem without a static information carrier and its mutation, freedom in variation for adaptation is also required as a characteristic of the system dynamics. Therefore, the system must not only have stability but also remain degrees of freedom to change. As mentioned above, if this change were completely free, the system would not be able to continuously retain or increase its information. Therefore, adaptability requires both strong constraints and a certain degree of freedom for changes as a property of the system dynamics, which is exactly homeorhesis (Figure 3B). Note that homeostasis and homeorhesis are not mutually exclusive properties. At least, homeorhesis in organisms and ecosystems would encompass random changes within the sublevels of their hierarchical structure. Consequently, homeorhesis may be considered a property of a higher hierarchy than homeostasis.

In systems ecology, it is postulated that systems at sub-organism levels in the hierarchies of living systems, such as organs or molecules, mainly demonstrate homeostasis, whereas super-organism levels, such as populations or ecosystems, mainly display homeorhetic properties [3]. For instance, molecular systems have an equilibrium or steady state they can maintain, whereas ecosystems are far from any equilibrium or steady state and continuously changing.

Organisms can play genetic evolution through natural selection without homeorhesis; however, a theoretical study has suggested that continuous evolution inevitably leads to the acquisition of homeorhesis [47]. More specifically, as a result of continuous evolutionary processes, the behavior of high-dimensional organisms was constrained to a small number of dominant mode dimensions, which correspond to the dimensions of proliferation rate. The changes due to steady fluctuations, responses to environmental changes (i.e., phenotypic plasticity for organisms), and long-term changes (evolution) are constrained into the dominant mode (Figure 3B). Therefore, for example, the direction of an adaptive change in the state space is predictable from the fluctuation, like a fluctuation-response relationship [48,49].

In this study, this hypothesis proposing the existence of such a dominant mode as a balance of strong constraints and a small degree of freedom is referred to as the Dominant Mode Hypothesis (DMH). The DMH demonstrates the emergence of homeorhesis and proposes that even if a genetic mutation is random, changes in the system are never random and fully controlled. This property depicts the adaptability of dynamics that facilitates ex ante adaptation rather than ex post adaptation as ordinarily considered in natural selection. Indeed, the DMH was experimentally tested using bacterial adaptation [47,50]. For instance, various types of environmental changes, such as osmotic pressure, high temperature, and starvation, seemed to induce similar changes in the transcriptome [51]. Similar observations have been made in proteomes under varying environmental conditions, including different nutrients and cultivation methods [47]. Additionally, it has been observed that responses to environmental changes and alterations resulting from adaptive evolution (laboratory evolution) were also similar [52]. Constraints to low-dimensional phenotypic states were further confirmed in a high-throughput laboratory evolution for various drug resistances [50].

Considering that ecosystems do not undergo clear natural selection, unlike living organisms, and are thought to exhibit stronger characteristics of homeorhesis rather than homeostasis [3], it can be proposed that the DMH may also be applicable to ecosystems. The DMH will be a powerful tool for predicting system changes, e.g., the response to the global warming of an ecosystem can be predicted from its daily fluctuations. Note that there is another useful idea of using the resilience of dynamical systems to describe changes in a steady state in a low-dimensional “efficient dimension” [53,54]. This idea focuses only on homeostasis and is fundamentally different from the DMH.

More generally, the DMH suggests an inevitable existence of strong dimensionality reduction for systems with adaptability, including all living systems. Living systems can fundamentally be viewed as dynamic networks comprising numerous dimensions. For instance, even in the bacterium *Escherichia coli*, there are over 1000 types of gene expression levels, in addition to the amount and spatial arrangements of many other molecules. From a control theory standpoint, managing scale-free networks, which typify biological networks, is exceedingly challenging [55]. However, it is posited that this difficulty is managed due to the interrelations among many dimensions and their constraint to fewer dimensions [56]. This dimensionality reduction has also been observed in the brain, which is a typical example of a biological information-processing system [57,58]. The dimensionality reduction *per se* would be an inherent feature of information-processing systems and may also appear as a topological constraint, such as biological “Bowtie” structures [59] or autoencoders in artificial neural networks [60]. Therefore, the DMH would be useful not only for understanding living systems with adaptability but also for constructing dynamic artificial information-processing systems as an application.

### 1.6. Using Experimental Ecosystems as a Phenomenological Approach

Despite numerous approaches to understanding the changes and stability of complex living systems, a comprehensive understanding at both macroscopic and microscopic levels remains elusive [61,62]. This gap indicates a potential shortfall in phenomenological approaches similar to those employed in thermodynamics.

Since living systems have many commonalities and strong universality, model experimental systems are useful. For example, the understanding gained with one of the simplest model organisms, *Escherichia coli*, has helped us understand many other organisms [63]. Such universality suggests common constraints and reduced dimensionality and, therefore, makes us expect that the systems can be described with a small number of macroscopic parameters.

In systems ecology, species-defined experimental ecosystems by synthetic assemblages of microorganisms, designated here as “synthetic ecosystems”, were proposed for experimental model ecosystems [64,65]. Experimental studies concerning ecosystem diversity, stability, and ecosystem services include outdoor systems such as Cedar Creek [66,67] and laboratory-level systems like Ecotron [68,69]. Even microcosms composed of microorganisms, despite their limited scale, are considered valuable for addressing global ecological issues [70]. However, assessing ecosystem stability poses various challenges, including issues related to replicability. Furthermore, exploring system characteristics sometimes requires studying conditions not present in natural ecosystems. For instance, in assessing a system’s resilience to external disturbances, it is crucial to impose various types of disturbances that are too extreme for the system’s continued existence. Additionally, to ascertain if the core characteristic of a sustainable ecosystem is “something”, it is vital to compare systems that vary only in the presence or absence of that “something,” ensuring that the system lacking this “something” cannot exist as a natural ecosystem.

To address these challenges in microcosms, we previously developed a high-throughput experimental system of a synthetic ecosystem consisting of only model microorganisms, comprising three important functional groups of the ecosystem: producers, decomposers, and consumers [71]. The synthetic ecosystem includes fundamental ecological processes like photosynthesis, predator–prey interactions, competition, and cooperation. Each species within this system is amenable to cryopreservation, ensuring experimental replicability. This experimental model ecosystem allows for systematic ecosystem experimentation under various conditions, including those unattainable in natural environments, akin to the role of *E. coli* as a model for various organisms.

Specifically, our model ecosystem facilitates the investigation of inter-ecosystem competition and the identification of ecosystem information carriers, as depicted in Figure 2, as well as the examination of ecosystem constraints in the form of homeorhesis, illustrated in Figure 3.

The mixture of two ecosystems shown in Figure 2B can be easily tested systematically in our model ecosystem. While such complete coalescence is unrealistic in macroscopic ecosystems, thereby lacking research, it is considered to be frequent in microbial ecosystems, known as “community coalescence” [72]. Experiments involving merged microbial communities demonstrated the strong influence of dominant species and support of dominant species by other species [73]. Therefore, these results suggested that the characteristics of dominant species, rather than diversity, are important for representing the system dynamics. The study represents an important step in investigating commonalities and discrepancies in various ecosystems; however, the tested experimental ecosystems lack predatory factors, a feature crucial in general ecosystems.

Moreover, it is also possible to address the DMH, i.e., homeorhesis and constraints of ecosystems, in our model ecosystem. The homeorhesis has been observed in the process of leading ecosystems towards stable states, called ecological succession, in an exceedingly simple synthetic ecosystem [74]. In similar synthetic ecosystems, it has also been demonstrated that stochastic fluctuations within the system adhere to a power law as a constraint [75]. Furthermore, in experimental ecosystems using more complex, field-collected microbial communities, even in microbial ecosystems with considerable population changes, the functional structure has been found to remain stable [76]. Similar experimental ecosystems have shown that even with changes in species, the overall phylogenetic structure is robust [77]. Those studies might suggest the existence of the homeorhesis and constraints in experimental ecosystems; however, the applicability of the DMH for ecosystems has not been empirically demonstrated. Experimentally demonstrating the DMH necessitates precise and numerous replicated experiments, a challenge in natural ecosystems or experimental ecosystems where high-throughput experimentation is arduous. Moreover, testing dimensionality reduction is unfeasible with an overly simplistic synthetic ecosystem. Nonetheless, our synthetic ecosystem possesses the potential to demonstrate the DMH as a high-throughput experimental system despite possibly having too few species. If the DMH is experimentally shown to be applicable to ecosystems, it will be of great help in proactive biodiversity conservation and ecosystem management.

Understanding ecosystem dynamics requires not only a macroscopic view but also microscopic insight, with a particular emphasis on comprehending evolution and population dynamics [78]. Microbial experimental systems are powerful tools for elucidating evolutionary processes, as demonstrated by many studies [79,80,81]. Our model ecosystem is also conducive to evolutionary research, and some pertinent results related to evolution are shown below, but this study will not delve deeply into discussions of evolution due to limited experimental data. In studies concerning evolution within similar synthetic ecosystems, numerous significant findings have been presented. For instance, evidence has been shown of species diversifying their survival strategies [82] and instances where free-living algae have shifted towards a more endosymbiotic existence [83,84]. These examples are being discussed from a broader perspective [85], suggesting experimentally that ecosystems tend toward an increase in information.

### 1.7. Experiments in This Study

We have demonstrated, using the model synthetic ecosystems, scenarios such as the coalescence of two ecosystems and the constraints of ecosystems shown in Figure 2 and Figure 3, respectively. Specifically, we quantified which ecosystem was more competitive by merging two ecosystems. The results showed that ecosystems with higher diversity were more competitively stable, and the species composition was more robust and had a higher ability to explain the dynamics than the population of dominant species that was flexible. Therefore, the scenario depicted in Figure 2C was valid in these experiments, and it was speculated that the information carrier and functional units of ecosystems were species composition, i.e., merged genomic information and species abundance, respectively. Moreover, we investigated the response to temperature changes and long-term changes in ecosystems. The results have suggested that the DMH is also applicable to ecosystems. While these results are not comprehensive enough to substantiate theories, those outcomes are appropriate for fostering discussion in this special issue and for demonstrating the potential for future research using our model synthetic ecosystem for connecting a wide range of living systems.

## 2. Materials and Methods

### 2.1. Microorganisms

This study used microcosms obtained in the previous study [71]; no individual microorganisms were prepared for this study. The materials and methods for preparing the microcosm used in this study have been described in detail in the previous study. The methods for this study were basically the same as the previous study, and a brief explanation is provided below.

### 2.2. Microcosm Experiments

For microcosm experiments, a 50 µL of culture solution was prepared in each well of a 384-well plate (#142761; Nunc, Rochester, NY, USA) and sealed with a heat-adsorption seal (#4ti-05481; 4titude, Wotton-under-Edge, UK). Liquid medium BG11HLB, which contains half the concentration of the BG-11 medium for cyanobacteria [86] and 1/100 the concentration of the LB medium for bacteria [87], was used for all experiments. The 384-well plates were placed on a white LED panel (TH-224X170SW; CCS, Japan) in an incubator at 23 °C with irradiation at an intensity of 50 μmol∙m^−2^∙s^−2^ for 12 h intervals. Different incubators with different temperatures were used for the experiments on temperature changes. The plate was shaken by inversion and spun down approximately twice a week. All ecosystems used in this study were passaged approximately once every two weeks with a 1/10 dilution into a fresh medium.

Specifically, in the ecosystem coalescence experiments, the origin of each microcosm was a 50 μL culture, which was subcultured biweekly for six months in the previous study [71]. To increase the volume for the coalescence experiments, each culture was replicated into eight separate 50 μL cultures during 10-fold dilution passages, followed by a two-week cultivation period. These eight cultures were then combined and further diluted 10-fold to prepare 2500 μL of each microcosm. Subsequently, each microcosm was allocated into 8 wells of a new 96-well plate, with 130 μL per well. There were eight different microcosms, each dispensed into 8 wells, thus occupying 64 wells in an 8 × 8 matrix. In this matrix, each horizontal row of eight wells represented one type of microcosm. Similarly, the microcosms were dispensed into another 96-well plate so that each vertical column of 8 wells contained the same microcosm. Using a 96-channel pipette, 115 μL from each well of these two 96-well plates was combined in a new 96-well plate, resulting in a total volume of 230 μL per well. This 8 × 8 matrix facilitated a comprehensive combination of two types of microcosms. On the diagonal, identical ecosystems were mixed. The wells along this diagonal represented two replicate experiments. Subsequently, 50 μL was transferred from each well into 4 wells of a new 384-well plate, yielding four replicates for each combination in the 8 × 8 matrix. The plate was sealed with a heat-adsorption seal and incubated at 23 °C. Subculturing was performed biweekly with a 10-fold dilution, as above.

Note that these experiments themselves do not directly correspond to the three types depicted in Figure 2C(i–iii). These experiments demonstrate only a single mixture process of interaction with neighboring ecosystems, where mixing occurs permanently (ii) or sometimes (iii), as represented in Figure 2C. Specifically, mixing was performed only at the initial step, and there was no interaction between the microcosms during subsequent passages, as described above. Suppose that, as presented in the Results and Discussion section below, the mixing of H and L types in Figure 2C results in a microcosm with diversity similar to or even greater than that of H. Conversely, it is presumed that H would become similar to L in the absence of interaction between microcosms, as in scenario (i), described earlier. Therefore, it is posited that diversity can only be maintained in situations akin to scenario (iii). Thus, it is important to note that while this experiment does not replicate scenarios (i), (ii), and (iii) in Figure 2C exactly, it is instrumental in testing the hypothesis outlined in the figure.

### 2.3. Measurements

A fluorescence plate reader (Varioskan Flash; Thermo, Waltham, MA, USA) was used for fluorescence spectroscopy to quantify the concentrations of Cyanobacteria, Chlorophyta, and *E. coli* (red fluorescent protein-labeled). For microscopy, each well was scanned using an inverted microscope (Nikon Ti-E with the Perfect Focus System and High Content Analysis; Nikon, Tokyo, Japan), and obtained bright-field images (two images with a time difference of 12 s) and fluorescent-field micrographs (three images with filter sets of Semrock FITC-3540C, Semrock TRITC-B, and Chroma 49006-ET-Cy5) of the center of each well (one position per well) from the bottom. A 60× objective lens (CFI S Plan Fluor ELWD ADL 60XC, Nikon, Japan) or 4× objective lens (CFI Plan Apochromat Lambda D 4X, Nikon, Japan), and a digital CMOS camera (Neo sCMOS, Andor, Belfast, UK; 2048 × 2048 pixels; 0.1 µm/pixel) was used for capturing images. For quantification of the concentration of *Tetrahymena thermophila*, the temporal variation of images obtained via low-magnification micrographs using the 4× objective lens, whose viewfield (one side is approximately 3.3 mm with a resolution of 1.6 µm/pixel) captured the entire well, because there was a correlation between *T. thermophila* concentration and the temporal variation since *T. thermophila* is large (major axis approximately 50 µm) and swims [71]. For the machine learning methods, the machine learning model that was constructed in the previous study was used [71]. The model was based on the publicly available object detection network framework YOLOv3 [88].

## 3. Results and Discussion

### 3.1. Ecosystems Used as Initial State

We prepared eight ecosystems, each with a certain degree of stability, and conducted coalescence experiments by mixing them pairwise, as detailed below. In the previous research, experimental ecosystems composed of a mix of 11 species were divided into 72 replicates and cultivated under identical conditions for six months. Stochastically, these ecosystems were separated into roughly seven patterns [71]. For this study, we utilized 8 of these 72 ecosystems. Out of the 11 species, 5 species could not survive in any of the ecosystems, leaving 6 species that persisted in at least across one of the seven patterns. These six species are listed in Table 1, and we refer to each of them by their abbreviated names shown in Table 1, i.e., Ecoli, Tetra, CyanoA, CyanoS, AlgaR, and AlgaC, in this study. These species include three important functional groups of ecosystems: producers, decomposers, and consumers. Some species exhibit mutualistic relationships, enabling their coexistence with multiple species, as they could not survive alone [71,89]. Moreover, Tetra had predator–prey interactions with Ecoli and CyanoS [90]. The producers include four species, with both prokaryotes and eukaryotes represented by two species each, which have potentially competitive relationships. Note that not all six species coexisted within a single ecosystem, but each ecosystem contained between two and five species.

**Figure 4:**
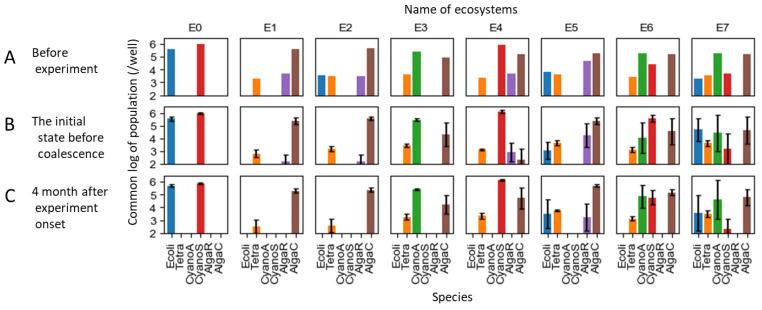
Population composition of the synthetic ecosystems utilized in this study. The numbers represent the count of individuals within an ecosystem volume of 50 μL. (**A**) The population composition of the original ecosystems in the previous study [71]. (**B**) The population composition at the initial state in this study. As an initial condition, we employed the logarithmic mean of eight values, combining the data at 0th (day 14) and 1st (day 28) transfers of four replicated wells. (**C**) The population composition after 4 months. Specifically, it presents the logarithmic mean of eight values, incorporating the 6th (day 102) and 7th (day 116) transfers. Error bars indicate the standard deviation of logarithmic values.

Figure 4 illustrates the species composition of the eight ecosystems used as the initial states (designated as Ecosystems E0 through E7). Figure 4A represents the original ecosystems, with 4B depicting the initial states that were achieved by diluting and aliquoting the original ecosystems into four replicates. Subsequently, the outcomes after approximately seven serial transfers, conducted approximately every two weeks, are shown in 4C (in other words, those that survived through 10^7^ to 10^8^ dilutions, roughly four months after the coalescence). While the ecosystems are stable overall, not all of them are entirely so. Specifically, the population of AlgaR tended to decrease gradually, and in some ecosystems, it fell below the detectable limit after four months. For the following analyses, the values from Figure 4B were utilized as the initial conditions, representing the states before coalescence.

### 3.2. Ecosystem Coalescence Experiments for Investigating Competitive Stability and Information Carrier

**Figure 5:**
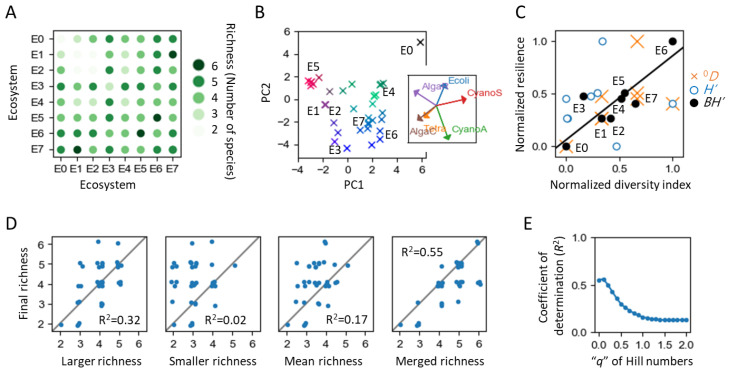
Results of the coalescence experiment. (**A**) Species richness in all 36 ecosystems following pairwise coalescence. For instance, the point where the horizontal axis is E2, and the vertical axis is E5, and vice versa, indicates the results of the mixture of ecosystems E2 and E5. The same results are displayed in both the upper right and lower left (N = 8). The diagonal line represents the results of mixing identical ecosystems (N = 4). (**B**) Results of the PCA. All 36 ecosystems are plotted. The inset shows the contribution of each species’ population to the PCA axes. (**C**) Relationship between *θ* and three diversity indexes. Both are linearly normalized so that the minimum is 0 and the maximum is 1. (**D**) Predicting the species richness at four months based on the species richness of the initial eight ecosystems. To avoid data overlap due to the discreteness, random values are added for visibility. “Merged richness” refers to the richness of a mixture of two ecosystems. In other words, it represents the gamma diversity of the two ecosystems, which is the same as the initial state of the merged ecosystem. There is a single point where the post-coalescence richness is higher than the merged richness of pre-coalescence ecosystems. This is because Figure 4B was used for the pre-coalescence state, and Ecoli was not detected in E2, but Ecoli may present below the detection limit. (**E**) q-dependence of predicting the post-coalescence Hill numbers based on the Hill numbers of merged populations of pre-coalescence ecosystems. The maximum value was 0.56 at *q* = 0.1, decreasing thereafter.

We mixed the above eight distinct ecosystems in a comprehensive pairwise manner, incorporating two of each, leading to an all-versus-all combination. Figure 5A illustrates the outcomes four months post-coalescence for these pairwise combinations. It encompasses the results of the 36 distinct ecosystems, considering both the _8_C_2_ combinations and the 8 original ecosystems. For the latter, identical ecosystems were mixed to align experimental conditions. Observationally, when ecosystems with lower and higher species richness (the number of species) were merged, the resulting species richness seemed to tend to gravitate towards the values of the higher species richness (see below for quantitative analyses).

Figure 5B represents the outcome of the 36 ecosystems after 4 months using a Principal Component Analysis (PCA) performed on the logarithm of the population sizes of the six species. The results for the unmixed eight ecosystems are indicated by text. An immediate observation is that the ecosystem consisting solely of prokaryotes (E0) was dramatically altered from its own state in every combination. Additionally, there appears to be a clustering toward ecosystems E4, E5, E6, and E7.

We investigated which ecosystems maintained their state stably. In this context, we introduce the concept of a competitive stability index (*Θ*) as a metric to assess the extent to which an ecosystem sustains its population composition post-coalescence. The competitive stability index of each *i*-th ecosystem is defined as $\Theta_{i}=1/\sum_{j}\sum_{k}{\left(x_{after,k,j}-x_{init,k,i}\right)}^{2}$, where *x*_after*,k,i*_ and *x*_init*,k,i*_ denote the logarithm of the population of species *k* in the *i*-th ecosystem at initial and 4 months, respectively. As *x* is a logarithm value, we used *x* = 0 for the population not detected. Figure 5C illustrates the relationship between *Θ* values and diversity indexes (α-diversity) of the eight ecosystems. We considered three simple measures of α-diversity: species richness (*^0^D*, the number of species), the Shannon–Wiener index (*H′*), and the biomass-corrected Shannon–Wiener index (*BH′*). In *BH′*, the probability of each species’ population (*p_i_*) in *H′* is substituted with the relative biomass of each species, which is the proportion of biomass represented by each species [91,92,93,94]. The values of approximate volume, shown in Table 1, were used as biomass values for each species.

The results indicated the highest correlation between *BH′* and *Θ*, with *R* = 0.86, *p* = 0.007, hereafter *α* = 0.05. Note that this relationship was somewhat influenced by the formulation of *Θ*. For instance, while species richness did not significantly correlate with *Θ* (*R* = 0.59, *p* = 0.12), its inverse (1/*Θ*) showed a significant negative correlation (*R* = −0.72, *p* = 0.04), similar to that of *BH′* (*R* = −0.73, *p* = 0.04). *BH′* normalizes the disparities between populations of larger and smaller organisms, making it closer to a measure of species richness.

Therefore, for a simple understanding, the larger the species richness, the more stable the ecosystem was, i.e., having better adaptability. These results support the mechanisms illustrated in Figure 2C that explain the sustainability or increase in ecosystem information. Note that the objective of this comparison of indicators is not to determine which is superior in representing natural ecosystems but to contrast the characteristics of stable ecosystems using straightforward indicators. For example, species richness, although deemed overly simplistic and problematic in depicting a natural ecosystem requiring estimation [20], has the benefit of involving just a single parameter, considerably fewer than other indicators. Therefore, if a phenomenon can be effectively explained by species richness, it is beneficial from an information criterion standpoint.

Conversely, *H′* failed to account for the competitive stability (*R* = 0.03, *p* = 0.95). This shortfall likely arises because *H′* inherently underrepresents species with larger biomass but smaller populations, thereby reducing their contribution. In systems ecology, larger individuals are often considered to carry more information [31], which is expressed in the opposite way in *H′*.

The obtained fact that ecosystems with a larger richness are more stable suggests that the larger richness of the two pre-coalescence ecosystems could more accurately predict the post-coalescence richness than the smaller one. However, it is not clear whether information from the ecosystem with smaller richness remains in the post-coalescence ecosystems. Using an analogy with organisms (Figure 2B, lower), it is necessary to clarify whether only information about ecosystem H remains, like predator–prey relationships or information that merges both ecosystems remains, like symbiosis.

We investigated which data set from the two ecosystems or their combination could better forecast the outcome. Specifically, we employed richness as the most fundamental indicator with the fewest number of parameters and determined how much the pre-coalescence richness could dictate the post-coalescence richness (Figure 5D). It was found that the larger richness had a greater coefficient of determination than the smaller or mean richness of the two ecosystems. This implies that the results are closer to the larger richness, consistent with the aforementioned competitive stability of ecosystems with higher richness. Additionally, the richness calculated from the merged two ecosystems (representing the gamma diversity of the two ecosystems, which is the same as the initial state of the merged ecosystem) had the greatest coefficient of determination. This suggests that the initial richness upon coalescence remains relatively unchanged, indicating that the information from the ecosystem with smaller richness was not lost but was influential in the resultant richness; thus, when comparing with the cases of organisms in the aforementioned Figure 2B, the observation that mixing two ecosystems results in the retention of information from both may suggest that the relationship between these ecosystems can be interpreted as not predatory but rather symbiotic.

In the results above, the richness was able to adequately explain the outcomes, whereas *H*’, i.e., population information, was less explanatory. This may differ from previous microbial coalescence studies where the dominant species could explain the outcomes [73]. Conversely, in a natural wetland ecosystem, it is known that an ecosystem state index NDVI (Normalized Difference Vegetation Index) can predict species richness more accurately than dominant populations [95], suggesting a potentially similar situation. To quantitatively verify this in our case, we employed an approach akin to the previous study of the natural wetland ecosystem [95], examining the predictability of outcomes by varying the order parameter q in the widely applicable diversity index known as Hill numbers Dq=∑ipiq1/(1−q), where *p_i_* is the proportion of individuals belonging to the *i*-th species. When *q* = 1, the formulation is undefined, but the mathematical limit as q approaches 1 is defined as D1=exp−∑ipilnpi, i.e., the exponential of *H′*.

Specifically, using a certain value of *q*, we calculated the *^q^D* from the population of two pre-coalescence ecosystems and used this as the explanatory variable, with the *^q^D* of the post-coalescence ecosystem after four months as the dependent variable to determine the coefficient of determination. This process was repeated with varying *q* values, and we obtained the *q* profile of the coefficient of determination (Figure 5E). Note that the interpretation of Hill numbers changes with the order parameter *q*. Roughly speaking, smaller *q* values emphasize the presence or absence of species, while larger values prioritize population sizes, i.e., the proportion of dominant species population in extreme cases. Specifically, *^0^D* equates to species richness, independent of population sizes. *^1^D* corresponds to the exponential of the Shannon–Wiener index, where population sizes are considered. *^2^D* equals the Simpson index, focusing more on the population sizes and highlighting the prevalence of dominant species.

The results show that the highest coefficient of determination was observed at a low *q* value of 0.1 (Figure 5E), indicating that species composition was robust and species abundance was flexible. They also satisfy, respectively, not functioning directly and functioning directly. Therefore, the information carrier and functional units of ecosystems were speculated as the species composition and species abundance, respectively. It is important to note that this speculation is based on a comparison between only these two aspects: species composition and species abundance. In reality, ecosystems comprise many other parameters.

This nature of flexibility of populations and robustness of species composition would be consistent with the characteristics of the human gut microbiota [96]. Moreover, this *q* profile is akin to the predictability of the mean NDVI in natural wetland ecosystems, maximum at *q* = 0.2 [95]. Therefore, our finding that the information carrier of ecosystems is species composition might be universally applicable to other ecosystems as well.

In our experimental system, the low explanatory power of the dominant species can be readily explained by the presence of predation. For instance, in a system consisting only of Ecoli and CyanoS, as represented in E0, both species exhibit small biomasses, leading to exceedingly high population numbers. When this ecosystem is mixed with one containing the predator, Tetra, the population of these smaller organisms diminishes rapidly. In the same sense, the predator is the largest in biomass; thus, their population is always small, but the outcome changes greatly depending on whether the predator is present or not, just like a keystone species [97]. Consequently, population size scarcely contributes as an explanatory variable. On the contrary, the rapid decrease in the prey population does not equate to extinction, and species often persist at low population levels, thereby maintaining species richness.

Thus, emphasizing the functions of each species can effectively elucidate the results, indicating the advantages of considering functional diversity. In our system, the species variety is too limited to be worth evaluating quantitatively, but conceptually, only ecosystem E0 lacks predators and may be deemed to have reduced functional diversity. Consequently, it might be inferred that ecosystems with greater functional diversity are more stable. Note that microbial experimental ecosystems in the previous study [73], where the dominant population shows high explanatory power, do not contain any predators, which may affect our results or the natural wetland ecosystem [95]. These ecosystems are, therefore, perceived as having low functional diversity, with competition occurring exclusively among them. Should a functional diversity index be uniformly applicable across all ecosystems, it might enable comparisons of markedly disparate ecosystems on an equal footing. While functional diversity presents various challenges due to its inherent complexity, ongoing enhancements aimed at ensuring universality and mathematical robustness are promising, positioning it as a potential comprehensive indicator [98]. Simultaneously, if our synthetic ecosystem were to be developed to include more species, it would become possible to experimentally demonstrate the advantages of functional diversity.

As mentioned above, the predator species Tetra plays an important role as a keystone species in this ecosystem. This keystone species is small in number and has a slow maximum rate of proliferation. The population was also robust for this predator species. These characteristics of robust, small in number, and slow are appropriate for an information carrier. For example, if the characteristics of a single individual of this keystone species change due to genetic variation, the characteristics of the whole ecosystem can change rapidly because the population size is small, and this species is influential. Although we did not compare this specific population with other parameters in this analysis, the keystone species itself might be the information carriers of ecosystems.

Our coalescence experiments consistently showed that species richness generally demonstrated robustness, thereby serving as an information carrier or a stable macroscopic parameter inherent to the systems. However, it is imperative to acknowledge that this finding does not universally apply to all ecosystems. The ecosystems utilized in the coalescence experiments here represent a recombination of divergent ecosystems that originated from the same source. Systematic investigations are essential to discern under what conditions certain parameters prove most useful or possibly appropriate as information carriers. In our synthetic ecosystems, this investigation is feasible, and further elucidation is expected from future research.

### 3.3. Ecosystem Constraints for Investigating the Dominant Mode Hypothesis

In this study, we experimentally investigated the DMH, which suggests that living systems possess a small degree of freedom by strong constraints, with changes predominantly confined to lower dimensions. Specifically, we examined the two types of ecosystem changes: (i) the rapid response of ecosystems in 7 days due to temperature changes and (ii) the gradual alterations of ecosystems observed in approximately 18 months without any induced environmental changes.

Before explaining our results, we describe the inherent limitations of these experiments below. Firstly, the measurements lack microscopic observation and rely solely on fluorometry using a plate reader (see Materials and Methods for details). While the precision of fluorometry is higher than that of data obtained from the microscopic observation, the dimensionality is limited, presenting a problem for the study of dimensionality reduction. Moreover, the low number of species of the synthetic ecosystem is also a significant problem. Nevertheless, we believe that presenting these results is beneficial as a trial demonstration for predicting ecosystem changes. For instance, the reduction from two dimensions to one can also be considered a kind of constraint.

We first tested environmental temperature changes. Specifically, for ecosystems initially at 23 °C, we varied the temperature to 25, 28, and 33 °C and observed the changes after seven days. The comparison of responses was not between the initial values and those after seven days, but between the responses at 23 °C after seven days and those at the varied temperatures after the same period because our ecosystems have a kind of stable state in the circumstances of subculturing every two weeks.

**Figure 6:**
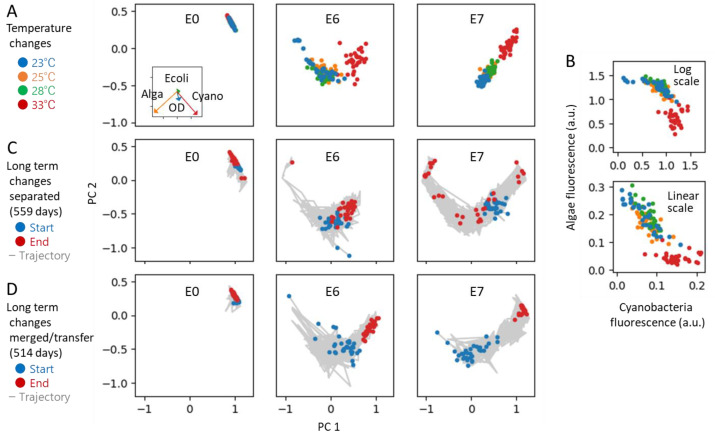
Constraints in ecosystem dynamics. (**A**) Alterations in response to temperature changes. (**B**) Results of fluorescence intensity in the temperature variation experiments. (**C**) Long-term changes without environmental changes imposed, when each of the 32 replicates was independent, for testing a situation like closed systems. (**D**) Long-term changes without environmental changes imposed when all 32 dispensed ecosystems were merged at every subculturing transfer.

We used three ecosystems: E0, the simplest ecosystem comprising only bacteria, and E6 and E7, ecosystems with the two largest richness among the eight types of ecosystems depicted in Figure 4. Figure 6A presents the results of PCA for the logarithm of the fluorescence intensity, projecting the results in two dimensions. In the case of ecosystem E6, the direction of fluctuation in the standard environment (23 °C, blue dots) appears to align with the response to temperature changes. E7 may adhere as well, suggesting changes within certain constraints. The simplest ecosystem, E0, exhibits little fluctuation and response change. This co-absence of fluctuation and response is also consistent with the implications of the DMH.

The reason why such constraints were observed was simple. First, examining the contribution fractions in PCA (as seen in the inset of Figure 6A), it is evident that only the two dimensions corresponding to cyanobacteria (Cyano) and green algae (Alga) are contributing, indicating that the PCA does not actually compress dimensions, unfortunately. Thus, the utilization of PCA here was merely for demonstration purposes, serving as an example for analyzing higher-dimensional ecosystems in future research. Nevertheless, the constraint from two dimensions to one was indeed present.

Second, Figure 6B illustrates the relationship between the fluorescence intensities representing the populations of cyanobacteria and green algae. These results suggest that the sum of both populations reaches a constant number as a carrying capacity, likely due to a trade-off resulting from competition for a resource such as carbon dioxide. Although this is a simplistic observation, it could be considered a typical constraint anticipated within ecosystems.

Next, we observed the long-term changes in the state of ecosystems. We branched each of the three aforementioned ecosystems into 32 replicates, continuing independent cultivation for 18 months. Although the fresh medium is supplied at each subculturing transfer, the biological elements are not supplied from outside each ecosystem, like the situation shown in Figure 2C(i). The results of the PCA, conducted in the same manner as in Figure 6A, are presented in Figure 6C. For both E6 and E7, the state transitions again roughly appear to align along a singular curve, a phenomenon explainable by the constraints in the dominant mode hypothesis. E0 again exhibited little changes.

We also tested the long-term changes when the 32 dispensed ecosystems were merged at every subculturing transfer (Figure 6D). This experiment tested a situation similar to the one shown in Figure 2C(ii). The results show the constraints as well.

All these results shown in Figure 6 suggest that the DMH is also applicable for ecosystems, which highlights the homeorhesis and adaptability of ecosystems. Although the results were poor, compression from two dimensions to one dimension was visible. However, as mentioned above, there are many problems with this experiment, and it is necessary to set better conditions and confirm it properly. At the same time, it is expected that similar analyses will be attempted in other experimental systems. In this study, replicate experiments were employed to account for the fluctuation of the ecosystem, but in natural ecosystems, utilizing daily fluctuations, for instance, could also be used. Our findings, exemplified by the trajectories in Figure 6C,D, suggest that the permissible direction of daily variations is constrained to a lower dimensionality.

Additionally, the results in Figure 6C,D, i.e., when closed and completely open situations, respectively, also show interesting results consistent with the scenario shown in Figure 2C. In Figure 6C, as closed systems, 32 replicates are scattered on the right edge, left edge, and center. The right and left edges indicated that producers were almost exclusively Cyano or Alga, respectively. One of them might actually be extinct. The plots between them indicate the states in which both Cyano and Alga coexist. Therefore, information on some of the 32 ecosystems decreased, as depicted in Figure 2C(i). In Figure 6D, the final results were almost entirely at the right edge. Thus, information on all 32 ecosystems, or more precisely, one large ecosystem, decreased, as depicted in Figure 2C(ii). These two results suggest that it is impossible for ecosystems to sustain or increase the information if they are completely closed or completely open, as shown in Figure 2C(i) and Figure 2C(ii), respectively, despite the fact that ecosystems with higher richness were more competitively stable as above.

## 4. Conclusions

In this study, we engaged with the significant question of how ecosystems change, discussing the information dynamics in living systems ranging from molecules to ecosystems from an ecosystem standpoint. Specifically, we utilized coalescence experiments in synthetic ecosystems to elucidate the quantitative relationship between biodiversity and competitive stability. We revealed that ecosystems with larger species richness were more stable in the disturbances by coalescence. Moreover, we found that species richness holds more robustness compared to population sizes in response to the process of coalescence, which was similar to a natural wetland ecosystem and the human gut microbiota. These results inferred that the information carrier of ecosystems was species composition or merged genomic information, and the functional unit of ecosystems was species abundance. The distinction between species composition and species abundance may be interpreted as a difference between species-level and individual-level parameters. Our experiments also suggested the potential applicability of the DMH to ecosystems, proposing that fluctuations in steady state, instantaneous responses to environmental changes, and long-term shifts are all constrained within the same lower dimensionality. These outcomes, combined with explanations of general aspects of adaptability, would contribute to the understanding and forecasting dynamics of not only ecosystems but also a wide range of living systems.

## Figures and Tables

**Table 1:** Species included in the synthetic ecosystems of this study.

	Species	Abbreviation	Classification	Functional Group	Shape, Approx. Vol.
#0	*Escherichia coli*	Ecoli	Proteobacteria	Decomposer	Rod, 1 μm^3^
#1	*Tetrahymena thermophila*	Tetra	Ciliophora	Consumer	Oval, 10^4^ μm^3^
#2	*Anabaenopsis circularis*	CyanoA	Cyanobacteria	Producer	Filamentous, 10^3^ μm^3^
#3	*Synechocystis* sp. 6803	CyanoS	Cyanobacteria	Producer	Spherical, 10 μm^3^
#4	*Raphidocelis subcapitata*	AlgaR	Chlorophyta	Producer	Crescent, 10^2^ μm^3^
#5	*Chlorella vulgaris*	AlgaC	Chlorophyta	Producer	Spherical, 10^2^ μm^3^

## Data Availability

Not applicable.
